# Neurologic alterations in an HIV adult patient with pertussis: a case report

**DOI:** 10.1186/s12879-020-05198-x

**Published:** 2020-07-02

**Authors:** María Camila Arango-Granados, Iván Mauricio Trompa

**Affiliations:** 1 Emergency Department, Fundación Valle del Lili, Cra 98 # 18 – 49, Cali, Valle del Cauca Colombia; 2 School of Medicine, Universidad Icesi, Cl. 18 # 122 - 135, Cali, Valle del Cauca Colombia; 3 IPS Universitaria León XIII, Infectology Service, Cl. 69 # 51C - 24, Medellín, Antioquia Colombia; 4 School of Medicine, Universidad de Antioquia, Cl. 67 # 53 -108, Medellín, Antioquia Colombia

**Keywords:** Whooping cough, Bordetella, Bronchopneumonia, Encephalopathy, Central nervous system infections, HIV, Acquired immunodeficiency syndrome

## Abstract

**Background:**

Pertussis is a highly contagious disease of public health interest caused by the bacterium *Bordetella pertussis*. Although its incidence has decreased substantially after the introduction of a vaccination, the burden of the disease remains high. Although the paroxysmal phase is highly disabling, complications are uncommon and more prevalent in children than in adults. The most frequent neurological complication is encephalopathy, but seizures, paresis, paraplegia, ataxias, aphasias, and decerebration postures have also been described. The complication of decerebration postures has not been previously reported in adults.

**Case presentation:**

We present a video case of an adult HIV patient with severe coughing paroxysms, post-tussive emesis and syncope, whose workup confirmed the diagnosis of a *B. pertussis* respiratory infection. During hospitalization, he had fluctuant encephalopathy and post-tussive decerebration postures following paroxysms. He was treated with antibiotic therapy and finally sent home without residual neurological deficits.

**Conclusion:**

This case illustrates the biological plausibility of neurologic complications of pertussis in adults, which, albeit rare, can cause important morbidities. Future research should explore whether there are differences in the clinical presentation, risk factors and pathophysiology of the disease among adults or interventions aimed at preventing or treating pertussis encephalopathy.

## Background

Pertussis is a highly contagious disease of public health interest caused by the bacterium *Bordetella pertussis*. Although its incidence has decreased substantially after the introduction of a vaccination, the burden of the disease remains high [1]. Neurologic complications are not common and have been predominantly described in children [2–4]. We present a video case of encephalopathy and post-tussive decerebration postures in an adult HIV patient with a confirmed diagnosis of *B. pertussis* respiratory infection. The patient provided written consent for publication of the information, images and videos related to his case.

## Case presentation

A 49-year-old man from Medellín (Colombia) was previously diagnosed with human immunodeficiency virus (HIV) infection and currently adhered to an antiretroviral therapy regimen; he had a undetectable viral load and a CD4+ cell count > 400 cells/mm^3^ at presentation. The patient complained of 4 weeks of initial dry cough, low fever, coryza, conjunctival injection and rhinitis. Two weeks after symptom onset, the cough became paroxysmal and severe, with post-tussive emesis and occasional post-tussive syncope (see video included as Additional file 1). No gastrointestinal or urinary symptoms were present. The patient denied being previously vaccinated with Tdap (tetanus toxoid, reduced diphtheria toxoid, and acellular pertussis vaccine). He commented that his daughter and granddaughter had mild upper respiratory infections days before his symptoms started.

**Additional file 1.** Coughing paroxysms in an adult patient with confirmed pertussis

**Fig. 1:**
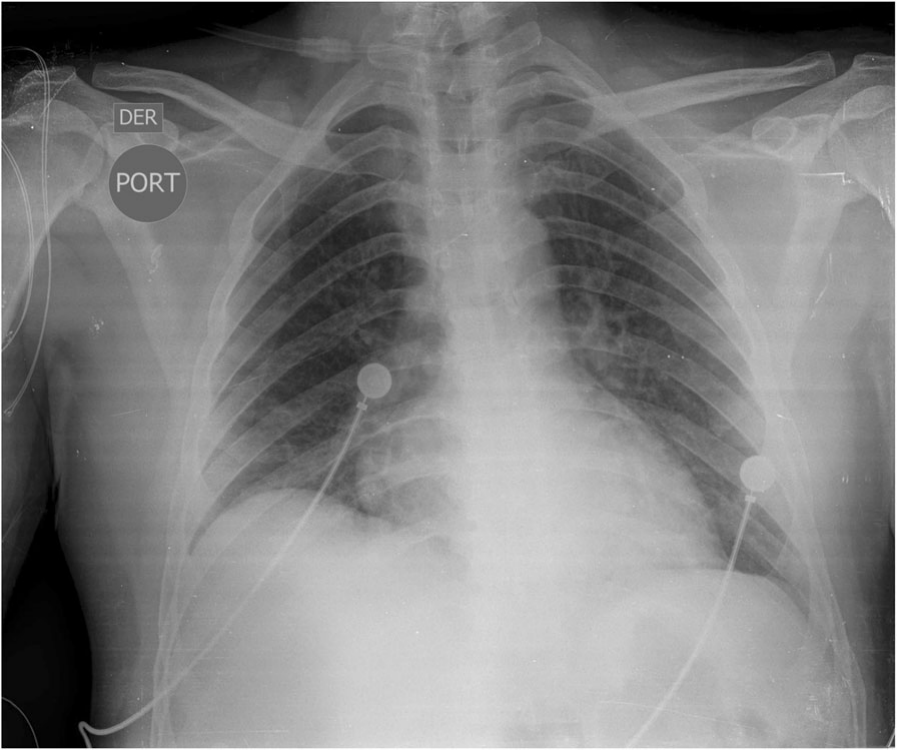
Chest X-ray acquired on admission of an adult patient with suspected pertussis

Upon admission, he had normal vital signs and no evidence of respiratory distress in the absence of cough. The laboratory results included a total leukocyte count of 13.030 cells/mm3 with 56% neutrophils and 31% lymphocytes, hypoxemia on arterial blood gases (oxygen partial pressure of 59 mmHg) and a lactic dehydrogenase of 232 U/L. The chest X-ray (Fig. 1) showed no clear evidence of alveolar occupation. Due to his past medical history, bronchoalveolar lavage (BAL) was performed, and samples were taken for gram staining, cultures and special stains. Polymerase chain reaction (PCR) for multiple respiratory pathogens was requested (BIOFIRE® FILMARRAY® Respiratory Panel RP2). This assay searches for 4 bacteria (*Bordetella pertussis* [detection of *ptxP*], *Bordetella parapertussis [*detection of IS*1001], Chlamydia pneumoniae* and *Mycoplasma pneumoniae*) and 18 viruses **(**adenovirus, coronavirus 229E [CoV-229E], coronavirus HKU1 [CoV-HKU1], coronavirus NL63 [CoV-NL63], coronavirus OC43 [CoV-OC43], human metapneumovirus [hMPV], human rhinovirus/enterovirus [HRV/EV), influenza virus A [FluA), influenza virus A H1 [FluA H1], influenza virus A H1–2009 [FluA H1–2009], influenza virus A H3 [FluA H3], influenza virus B [FluB], parainfluenza virus 1 [PIV1], parainfluenza virus 2 [PIV2], parainfluenza virus 3 [PIV3], parainfluenza virus 4 [PIV4], respiratory syncytial virus [RSV] and Middle East respiratory syndrome coronavirus) [5].

Because of the chronicity of the cough, a computed tomography (CT) scan of the paranasal sinuses was performed and revealed mucous thickening of all the sinuses due to chronic inflammation. Symptomatic management was initiated for chronic sinusitis, and clarithromycin was empirically started for a suspected pertussis-like syndrome. A day after admission, PCR for multiple respiratory pathogens confirmed the presence of *B. pertussis*. No other microorganisms were identified through this method. Other confirmation tests for *B. pertussis* were not performed given the 99–100% specificity of PCR [6] and the low yield of positive results with serology or culture techniques [7]. Common aerobic respiratory pathogens were not identified in the BAL cultures. Other opportunistic infections were excluded by special stains and laboratory tests on admission.

During his hospitalization, he continued to experience frequent paroxysmal cough (9 to 12 per day), with prolonged apnea, syncope and desaturation during the episodes. Additionally, his wife described that during the episodes his eyes deviated upwards, his arms became rigid while flexing his wrists, with subsequent myoclonic movements of both arms and legs (see video included as Additional file 2). The patient occasionally had diminished alertness and bradypsychia between coughing paroxysms. A careful evaluation excluded potential toxins or drug ingestion. In addition, the clear relationship between the coughing paroxysms and periods of full recovery without any neurological abnormalities precluded the need for lumbar puncture or neuroimaging. He finished a 7-day course of clarithromycin without requiring ventilatory support and without residual neurological deficits. He was finally sent home with persistent coughing paroxysms, although less severe and less frequent.

**Additional file 2.** Encephalopathy and decerebration postures in an adult patient with confirmed pertussis

## Discussion and conclusion

Pertussis or “whooping cough” is an acute respiratory disease caused by the gram-negative cocobacillus *B. pertussis*. This bacterium is a strict human pathogen that is transmitted through respiratory droplets. The disease is highly contagious [8]. There are nine recognized *Bordetella* species, but in addition to *B. pertussis,* only 3 of them have been associated with respiratory infections in humans: *B. bronchiseptica, B. parapertussis*, and *B. holmesii* [9]. This disease is more frequent in children than in adults, but there has been a recent increase in the incidence of the disease in previously vaccinated adults, possibly due to waning vaccine-induced immunity [2]. After the implementation of a vaccination in the 1940s, the incidence and mortality of pertussis were reduced by 92 and 99%, respectively [10]. However, there are still approximately 48.5 million cases per year, with up to 295,000 deaths attributed to the disease [1].

The course of the disease can be described by 3 phases. The first phase is the catarrhal phase, which lasts approximately 1–2 weeks and is indistinguishable from other upper respiratory infections. However, in adults, excessive lacrimation and conjunctival injection can raise suspicion of pertussis [11]. In the next phase, the cough turns paroxysmal and is accompanied by inspiratory whooping that gives name to the disease. This, however, is a phenomenon rarely observed in adults. The presence of post-tussive emesis and post-tussive syncope is characteristic [2]. This phase can last between 2 weeks and 2 months [11, 12]. Finally, during the convalescent phase, the cough gradually decreases in intensity, although it may be present for up to 2 months [9, 11].

Other microorganisms other than *B. pertussis* can produce a pertussis-like syndrome. The most frequently identified pathogens are rhinovirus/enterovirus (32.9%), respiratory syncytial virus (31.4%), parainfluenza virus (30.7%), coronavirus (11.4%), adenovirus (5.0%), *Mycoplasma pneumoniae* (2.9%), *Chlamydia pneumoniae* [13], *B. parapertussis* and *B. holmesii* [9].

Diagnostic suspicion of pertussis is rare in adults, especially since the presentation is not usually as classic as that in children. Paroxysmal cough in adults, for example, has a likelihood ratio (LR) + of 1.2 (1.04–1.7) and an LR - of 0.61 (0.52–0.72), which demonstrates its limited diagnostic utility [14]. Post-tussive vomiting has a similar diagnostic performance, with an LR + of 1.7 (1.3–2.3) and an LR - of 0.83 (0.73–0.93) [14]. Finally, classic whooping cough in adults is very rare, with a diagnostic sensitivity ranging from 16 to 28% [14].

PCR is an important tool for timely diagnosis of pertussis and is increasingly available to clinicians. There are several assays available that target different genetic sequences and therefore have different diagnostic performances. QIAstat-Dx RP, for example, targets the multicopy insertion sequence (IS481) that is present in multiple Bordetella species (*B. pertussis, B. holmesii, and B. bronchiseptica*) [15]. The BIOFIRE® FILMARRAY® Respiratory Panel used in this case is designed to be specific for the detection of B. pertussis and targets the single-copy promoter region of the pertussis toxin gene (*ptxP*). Although the greater specificity (99.3%) of the latter is accompanied by lower sensitivity [16, 17], a positive result supports the diagnosis of pertussis.

Complications are not common in adolescents and adults. However, cases of angina, carotid dissection, intracranial hemorrhage and fractures have been reported [2]. Encephalopathy is also a rare complication [3] that usually appears in the first 2 to 4 weeks after the onset of cough [4]. This complication occurs more frequently in nonvaccinated children < 2 months, although it has also been described in adults [18]. The most frequent clinical manifestation is the onset of seizures, but paresis, paraplegia, ataxias, aphasias and decerebrating postures have also been described. The reason is believed to be penetration of *Bordetella spp.*-specific antigens to the central nervous system [3]. High levels of pertussis toxin (PT) and filamentous hemagglutinin (FHA) antibodies have been identified in the cerebrospinal fluid of these patients [3]. However, hypoxic encephalopathy secondary to apneas is also a possible cause of these manifestations [9].

To the best of our knowledge, this is the first reported case of a decerebrating posture caused by pertussis in an adult. It is unknown if there are differences in the clinical presentation, risk factors and pathophysiology of the disease among adults or if there are interventions aimed at preventing or treating pertussis encephalopathy. This case illustrates the biological plausibility of neurologic complications of pertussis in adults, which, albeit rare, can cause important morbidities.

Several antibiotics have in vitro activity against B. pertussis [19–21]. The first-line treatment of *B. pertussis* is macrolides, and oral erythromycin has been the mainstay of treatment over the past 30 years [9]. Antibiotics during the catarrhal stage appear to shorten the duration of symptoms and, in most cases, clear the organism from the upper respiratory tract within 5 days of the initiation of therapy [22, 23].

Erythromycin, a macrolide antibiotic, has been the antimicrobial of choice for treatment or postexposure prophylaxis of pertussis. It is usually administered in 4 divided daily doses for 14 days. Since erythromycin is accompanied by uncomfortable to distressing side effects that result in poor adherence to the treatment regimen two other macrolide agents (azithromycin and clarithromycin) are used for the treatment and prophylaxis of pertussis [23]. An alternate regimen is trimethoprim-sulfamethoxazole [24].

Antibiotic therapy appears to be the most effective in the first 21 days after the onset of symptoms. After this time, there is uncertainty about the usefulness of antibiotics to improve the clinical outcomes [25]. Otherwise, management is generally symptomatic. However, there is doubtful evidence about the usefulness of diphenhydramine, dexamethasone, salbutamol or pertussis immunoglobulins in reducing coughing paroxysms. These interventions have still no proven efficacy in reducing the frequency of vomiting, frequency of whooping cough, frequency of cyanosis, development of serious complications, long hospital stays or mortality from any cause [26].

In severe cases, admission to the intensive care unit and invasive mechanical ventilation are required. Even extracorporeal membrane oxygenation and leukoreduction have been used for the management of pulmonary hypertension and cardiogenic shock secondary to the marked hyperleukocytosis that may accompany this disease [27, 28]. However, the usefulness of leukoreduction or plasma exchange therapies is still a matter of debate.

The case presented was not accompanied by hyperleukocytosis, which is observed in some severe cases [29–31]. A probable explanation is the history of HIV infection; however, while current reports of B. pertussis and HIV infection describe the clinical characteristics of the disease [32, 33], the impact on the white blood cell count is unknown.

In conclusion, pertussis is a highly contagious disease of public health interest caused by the bacterium *Bordetella pertussis*. Although its incidence has decreased substantially after the introduction of a vaccination, the burden of the disease remains high. Although the paroxysmal phase is highly disabling, complications are uncommon and more prevalent in children than in adults. The most frequent neurological complication is encephalopathy, but seizures, paresis, paraplegia, ataxias, aphasias, and decerebration postures have also been described. To the best of our knowledge, this is the first reported case of a post-tussive decerebration posture caused by pertussis in an adult, which confirms the biological plausibility of neurologic complications of pertussis in this population. Future research should explore whether there are differences in the clinical presentation, risk factors and pathophysiology of the disease among adults or interventions aimed at preventing or treating pertussis encephalopathy.

## Data Availability

The datasets used and/or analysed during the current study are available from the corresponding author on reasonable request.

## Abbreviations

BAL: Bronchoalveolar lavage
PCR: Polymerase chain reaction
CT: Computed tomography
PT: Pertussis toxin
FHA: Filamentous hemagglutinin

## References

[1] Crowcroft NS, Stein C, Duclos P, Birmingham M (2003). How best to estimate the global burden of pertussis?. Lancet Infect Dis.

[2] Cornia PB, Hersh AL, Lipsky BA, Newman TB, Gonzales R (2010). Does this coughing adolescent or adult patient have pertussis?. JAMA..

[3] Grant CC, McKay EJ, Simpson A, Buckley D (1998). Pertussis encephalopathy with high cerebrospinal fluid antibody titers to pertussis toxin and filamentous hemagglutinin. Pediatrics..

[4] Zellweger H (1959). Pertussis encephalopathy. Arch Pediatr.

[5] Leber AL, Everhart K, Daly JA, Hopper A, Harrington A, Schreckenberger P, et al. Multicenter Evaluation of BioFire FilmArray Respiratory Panel 2 for Detection of Viruses and Bacteria in Nasopharyngeal Swab Samples. J Clin Microbiol. 2018;56(6):e01945–17. 10.1128/JCM.01945-17 PMC5971546 29593057

[6] Lee AD, Cassiday PK, Pawloski LC, Tatti KM, Martin MD, Briere EC (2018). Clinical evaluation and validation of laboratory methods for the diagnosis of Bordetella pertussis infection: culture, polymerase chain reaction (PCR) and anti-pertussis toxin IgG serology (IgG-PT). PLoS One.

[7] Gilberg S, Njamkepo E, Du Châtelet IP, Partouche H, Gueirard P, Ghasarossian C (2002). Evidence of Bordetella pertussis infection in adults presenting with persistent cough in a french area with very high whole-cell vaccine coverage. J Infect Dis.

[8] Cherry JD, Heininger U (2004). Pertussis and other Bordetella infections. Textbook of pediatric infectious diseases.

[9] Mattoo S, Cherry JD (2005). Molecular pathogenesis, epidemiology, and clinical manifestations of respiratory infections due to Bordetella pertussis and other Bordetella subspecies. Clin Microbiol Rev.

[10] Roush SW, Murphy TV, Group V-PDTW (2007). Historical comparisons of morbidity and mortality for vaccine-preventable diseases in the United States. JAMA..

[11] Paisley RD, Blaylock J, Hartzell JD (2012). Whooping cough in adults: an update on a reemerging infection. Am J Med.

[12] Yaari E, Yafe-Zimerman Y, Schwartz SB, Slater PE, Shvartzman P, Andoren N (1999). Clinical manifestations of Bordetella pertussis infection in immunized children and young adults. Chest..

[13] Saiki-Macedo S, Valverde-Ezeta J, Cornejo-Tapia A, Castillo ME, Petrozzi-Helasvuo V, Aguilar-Luis MA (2019). Identfication of viral and bacterial etiologic agents of the pertussis-like syndrome in children under 5 years old hospitalized. BMC Infect Dis.

[14] Ebell MH, Marchello C, Callahan M (2017). Clinical diagnosis of Bordetella pertussis infection: a systematic review. J Am Board Fam Med.

[15] Leber AL, Lisby JG, Hansen G, Relich RF, Schneider UV, Granato P, et al. Multicenter Evaluation of the QIAstat-Dx Respiratory Panel for Detection of Viruses and Bacteria in Nasopharyngeal Swab Specimens. J Clin Microbiol. 2020;58(5):e00155–20. 10.1128/JCM.00155-20 PMC7180242 32132186

[16] Jerris RC, Williams SR, MacDonald HJ, Ingebrigtsen DR, Westblade LF, Rogers BB (2015). Testing implications of varying targets for Bordetella pertussis: comparison of the FilmArray respiratory panel and the focus B. pertussis PCR assay. J Clin Pathol.

[17] Tatti KM, Wu KH, Tondella ML, Cassiday PK, Cortese MM, Wilkins PP (2008). Development and evaluation of dual-target real-time polymerase chain reaction assays to detect Bordetella spp. Diagn Microbiol Infect Dis.

[18] Halperin SA, Marrie TJ (1991). Pertussis encephalopathy in an adult: case report and review. Rev Infect Dis.

[19] Hoppe JE, Haug A (1988). Antimicrobial susceptibility of Bordetella pertussis (part I). Infection..

[20] Hoppe JE, Eichhorn A (1989). Activity of new macrolides against Bordetella pertussis and Bordetella parapertussis. Eur J Clin Microbiol Infect Dis.

[21] Hoppe JE (1998). State of art in antibacterial susceptibility of Bordetella pertussis and antibiotic treatment of pertussis. Infection..

[22] Bergquist SO, Bernander S, Dahnsjö H, Sundelöf B (1987). Erythromycin in the treatment of pertussis: a study of bacteriologic and clinical effects. Pediatr Infect Dis J.

[23] Tiwari T, Murphy TV, Moran J (2005). National Immunization Program CDC. Recommended antimicrobial agents for the treatment and postexposure prophylaxis of pertussis: 2005 CDC Guidelines. MMWR Recomm Rep.

[24] Hoppe JE, Halm U, Hagedorn HJ, Kraminer-Hagedorn A (1989). Comparison of erythromycin ethylsuccinate and co-trimoxazole for treatment of pertussis. Infection..

[25] Bennett J, Dolin R, Blaser M (2020). Mandell, Douglas, and Bennett's principles and practice of infectious diseases.

[26] Bettiol S, Wang K, Thompson MJ, Roberts NW, Perera R, Heneghan CJ (2012). Symptomatic treatment of the cough in whooping cough. Cochrane Database Syst Rev.

[27] Rowlands HE, Goldman AP, Harrington K, Karimova A, Brierley J, Cross N (2010). Impact of rapid leukodepletion on the outcome of severe clinical pertussis in young infants. Pediatrics..

[28] Domico M, Ridout D, MacLaren G, Barbaro R, Annich G, Schlapbach LJ (2018). Extracorporeal membrane oxygenation for pertussis: predictors of outcome including pulmonary hypertension and Leukodepletion. Pediatr Crit Care Med.

[29] Heininger U, Klich K, Stehr K, Cherry JD (1997). Clinical findings in Bordetella pertussis infections: results of a prospective multicenter surveillance study. Pediatrics..

[30] Hodge G, Hodge S, Markus C, Lawrence A, Han P (2003). A marked decrease in L-selectin expression by leucocytes in infants with Bordetella pertussis infection: leucocytosis explained?. Respirology..

[31] Carbonetti NH. Pertussis leukocytosis: mechanisms, clinical relevance and treatment. Pathog Dis. 2016;74(7):ftw087. 10.1093/femspd/ftw087 PMC5761200 27609461

[32] Nunes MC, Downs S, Jones S, van Niekerk N, Cutland CL, Madhi SA (2016). Bordetella pertussis infection in south African HIV-infected and HIV-uninfected mother-infant dyads: a longitudinal cohort study. Clin Infect Dis.

[33] du Plessis NM, Ntshoe G, Reubenson G, Kularatne R, Blumberg L, Thomas J (2018). Risk factors for pertussis among hospitalized children in a high HIV prevalence setting, South Africa. Int J Infect Dis.

